# The Effect of N-Acetyl-Cysteine on *NRF2* Antioxidant Gene Expression in Asthenoteratozoospermia Men: A Clinical Trial Study

**DOI:** 10.22074/ijfs.2020.44411

**Published:** 2020-10-12

**Authors:** Rahil Jannatifar, Kazem Parivar, Nasim Hayati Roodbari, Mohammad Hossein Nasr-Esfahani

**Affiliations:** 1 Department of Biology, Science and Research Branch, Islamic Azad University, Tehran, Iran; 2 Department of Reproductive Biotechnology, Reproductive Biomedicine Research Center, Royan Institute for Biotechnology, ACECR, Isfahan, Iran; 3 Isfahan Fertility and Infertility Centre, Isfahan, Iran

**Keywords:** Factor Erythroid 2-Related Factor 2, Nuclear Asthenoteratozoospermia, N-Acetyl-Cysteine, Oxidative Stress

## Abstract

**Background:**

One of the important factor associated with male infertility is high production of reactive oxygen species (ROS). The main function of Nuclear factor erythroid 2-related factor 2 (*NRF2*) is to activate the cellular anti- oxidant response by inducing the transcription of a wide array of genes that can combat the harmful effects of factors such as oxidative stress. The purpose of this study was to evaluate the effect of N-acetyl-L-cysteine (NAC), as an antioxidant drug, on NRF2 Gene Expression in Asthenoteratozoospermia Men.

**Materials and Methods:**

In this randomized, blinded clinical trial study, included 50 infertile men with asthenoteratozoo- spermia, who received NAC (600 mg, three times daily). Sperm parameters analyzed according to the world health organiza- tion (WHO; 2010). Sperm DNA fragmentation, relative *NRF2* expression, and seminal plasma level of antioxidant enzymes were measured by TUNEL assay, reverse transcription polymerase chain reaction (RT-PCR) and ELISA test, respectively.

**Results:**

After NAC treatment, findings showed a significant increase in sperm concentration and motility compared to pre-treatment status, whereas the percentage of abnormal morphology and DNA fragmentation was significantly decreased (P<0.05). A significant improvement in expression of *NRF2* gene and antioxidant enzyme levels were ob- served compared to pre-treatment by NAC (P<0.05). Significant correlations were observed between NRF2 mRNA expression level, specific sperm parameters and level of antioxidant enzymes (P<0.05).

**Conclusion:**

The results demonstrated that NAC oral supplementation protected against oxidative stress by enhancing *NRF2* expression. This could improve semen parameters quality parameters in asthenoteratozoospermia men (Regis- tration number: IRCT20170830035998N4).

## Introduction

One of the main causes of infertility in men is oxidative stress or high production of reactive oxygen species (ROS). It can also be provoked from reduced antioxidant capacity of semen and spermatozoa creating the conditions termed oxidative stress (1). Oxidative stress contributes to damage to various sperm parameters such as sperm morphology, sperm count and sperm DNA fragmentation associated with reducing fertility (2). Although, low amounts of ROS is essential for physiological and functional processes (such as acrosome reaction, capacitation and perm-oocyte penetration), excessive production of ROS can negatively impact the sperm quality and subsequently hampers fertility (3). Naturally, excessive production of ROS is counterbalanced by enzymatic and non-enzymatic antioxidants present in male reproductive tract (4). Production of antioxidant enzymes are regulated by a common regulatory factor-like nuclear factor erythroid 2-related factor 2 (*NRF2*) (5). *NRF2* regulates gene transcriptions containing antioxidant response elements (AREs) (6) like catalase (CAT), superoxide dismutase (SOD), and glutathione peroxidase (GPX).

In normal conditions, NRF2 is repressed by the negative regulator protein Keap1, largely localized in the cytoplasm. In this condition, *NRF2* is targeted by ubiquitination and proteasome degradation. Under oxidative stress condition *NRF 2* is phosphorylated. This phenomenon disrupts formation of the Keap1- *NRF 2* complex. Subsequently, *NRF2* is translocated in the nucleus and the level of enzymes containing this regulatory element is up-regulated (7).

NAC is derived from amino acid L-cysteine containing sulfhydryl groups that has free radical scavenging activ ity (8-10). Therefore, it is supplemented to alleviate glutathione (GSH) depletion during oxidative stress. Despite the well-known antioxidant capacity of NAC in different oxidative stress conditions (including male infertility) the correlation between NAC-induced oxidative protections and signaling transduction pathway remains to be elucidated (11-12).

Therefore, we investigated expression of *NRF2* in the sperm of asthenoteratozoospermia individuals treated with NAC. In addition, we studied relationship of *NRF2* expression with protein level of antioxidant enzymes, including CAT, SOD and GPX.

## Materials and Methods

A randomized, blinded clinical trial was designed for this study. A total of 50 infertile men with idiopathic asthenoteratozoospermia, at the age of 25 to 40 years old, were enrolled. Patients were referred to ACECR Infertility Research Center (Qom, Iran) from July 2018 to November 2018. None of the infertile couples had previously achieved pregnancy.

Inclusion criteria were infertile men with no history of varicocele, obstruction, cancer and chemotherapy as well as abnormal testes, leukospermia, cigarette smoking and alcohol consumption. Infertile patients were considered as male individuals with “asthenoteratozoospermia”, according to the world health organization (WHO) guidelines (13). A normal female partner was defined as a woman with regular menses, normal hormonal profile and hysterosalpigogram. The male individuals were defined as asthenozoospermic, if their total sperm motility was below 40% and/or their progressive motility was below 32%. Most of our participants had absolute asthenozoospermia and both parameters were below the WHO criteria. During this study, the patients received NAC (600 mg daily, for three months). Variables sperm parameters, DNA fragmentation index, *NRF2* gene expression and level of the antioxidant enzymes in seminal plasma were measured before and after intervention.

### Semen analysis and preparation

Sperm analysis was performed according to the WHO guidelines criteria, 2010 (14). All Semen samples were collected by masturbation after 3-4 days of abstinence and allowed to liquefy for 15-30 minutes at room temperate. Total and progressive motility were analyzed using the computer-aided sperm analysis (CASA) system (LABOMED, SDC313B, and Germany). Sperm morphology was stained with Papanicolaou and 200 sperms were evaluated per slide (15). Sperm number was counted by a sperm counting chamber and expressed as million/ ml. Samples with more than 1 million leukocytes in 1 ml of semen were excluded from the study. Semen samples were washed by Ham’s F-10 solution. The resulting sperm pellet was divided into several aliquot parts and they were kept frozen at -80˚C for subsequent analyses of RNA and biochemical factor levels.

### Assessment of DNA fragmentation (TUNEL assay)

Sperm DNA fragmentation analysis was determined using the in-situ cell death detection kit (Roche, Germany) based on the labeling of DNA strand breaks (TUNEL technology) (16). At least 200 stained sperms per field were assessed under an epifluorescent microscope (BX51, Olympus, Japan) at ×100 magnification. Percentage of the sperms with DNA-damaged was considered as number of TUNEL-positive (green fluorescence) and percentage of the sperms with intact DNA was considered as number of TUNEL-negative (red fluorescence).

### Assessment of *NRF2* by reverse transcription–polymerase chain reaction

After complete liquefaction, the cells in 1 ml of every sample were pelleted by centrifugation (6000 rpm). Total cellular RNA extraction was performed by using RNeasy Plus Micro Kit (Qiagen, Germany) according to the manufacturer’s instruction.

To remove DNA contamination, the extracted RNA samples were treated with DNase I. cDNA was reverse transcribed from 2μg of total RNA using M-MLV reverse transcriptase (Fermentase Corporation, Lithuania) and the corresponding oligonucleotide primers. Polymerase chain reaction (PCR) was carried out using 2μg cDNA specific primers for the both *GAPDH* and *NRF2* genes (Table 1).

**Table 1:** Primers used for RT-PCR analysis


Transcript	Sequence (5'-3')	Length of DNA product (bp)

*GAPDH*	F:TGGCTACAGCAACAGGGTG	104
	R: CTCTTGTGCTCTTGCTGGG	
*NRF2*	F:AGCACATCCAGTCAGAAACC	203
	R:TAGCCGAAGAAACCTCATTG	


Real-time PCR program consisted of enzyme activation at 95°C for 30 seconds, followed by 40 cycles of a twostep program, including template denaturation at 95°C (5 seconds) and annealing/extension at 58°C (30 seconds). The PCR product sizes were 203bp for *NRF 2* and 104bp for GAPDH. The 2^-∆∆Ct^ method was calculated to represent the relative quantification of mRNA expression of NRF2 after normalization to that of GAPDH, where ∆CT= (CT, NRF2 antioxidant genes-CT, *GAPDH*).

### Assessment of semen biochemical factors

For the biochemical factors analysis, we separated seminal plasma and stored it at -80°C until use. Total antioxidant capacity (TAC) and Malondialdehyde (MAD) of the plasma for all samples were measured using the commercial kits (Zell Bio GmbH, Wurttemberg and Germany). The level of superoxide dismutase (SOD), catalase (CAT) and glutathione peroxidase (GPX) was assessed by ELIZA kit (Abnova Corporation, Taiwan).

### Statistical analysis

The statistical software SPSS (Version 20, USA) was used for data analysis. Data are presented as mean ± standard error of the mean (SEM). The paired sample t-test was used for comparison of the samples before and after NAC treatment. Correlation between different variables was studied using the Pearson correlation coefficient. A P<0.05 was considered statistically significant.

### Ethical considerations

This clinical trial study was registered in the Iranian Registry of clinical trials (Registration number: IRCT20170830035998N4) and it was approved by the Ethics Committee for Research Involving Human Subjects at Science and Research Branch of Azad Medical University (Tehran, Iran). An informed consent was obtained from each participant and this study was in continuation of previous study (17).

## Results

### Effect of N-acetyl-L-cysteine treatment on sperm parameters

Sperm concentration, sperm motility (total and progressive motilities), sperm morphology were significantly different at end of the study (Fig . 1). After NAC supplementation, mean sperm concentration and percentage of motile sperm were significantly increased compared to the samples before NAC treatment (P<0.05). The results showed significant improvement in the samples with abnormal morphology (P<0.05). Additionally, significant improvement was observed in sperm DNA fragmentation after treatment by NAC (P<0.01).

**Fig.1:**
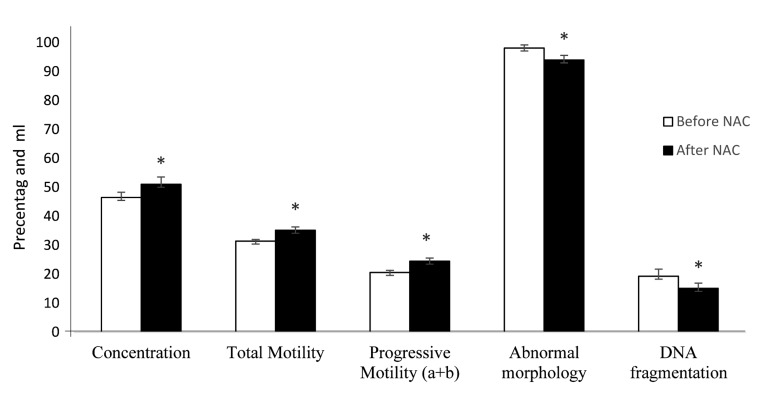
Comparison of sperm parameters before and after NAC treatment. *; significant difference before and after treatment, and NAC; N-acetyl-cysteine.

### Effect of N-acetyl-L-cysteine treatment on NRF2 gene expression

**Fig.2:**
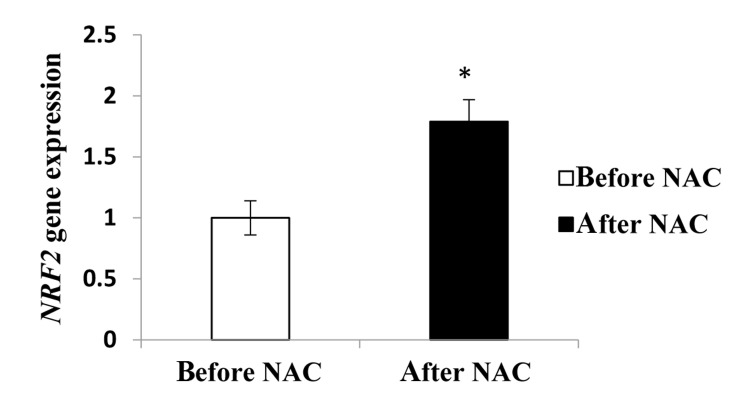
Comparison of relative expression of NRF2 before and after NAC treatment. NAC; N-acetyl-cysteine, NRF2; Nuclear factor erythroid 2-related factor 2, and *; Significant difference before and after NAC treatment.

To explore role of NAC in regulating the expressions of NRF2, we analyzed relative expression of NRF2 gene in sperm cells using RT-PCR method. As shown in Figure 2, expression of NRF2 gene after treatment was significantly higher than before treatment. The results indicated that after intervention, NAC significantly increased NRF2 expression level (1.00 ± 0.14 vs. 1.79 ± 0.18 respectively, P=0.01).

### Effect of N-acetyl-L-cysteine tr eatment on biochemical factors

A higher level of TAC on seminal plasma was observed after NAC supplementation. Moreover, the level of MDA on seminal plasma was significantly lower in infertile men after treatment with NAC compared to with before treatment with NAC (P<0.05). In addition, the results demonstrated that CAT, GPX and SOD levels were significantly increased in NAC treated group (P<0.05, Table 2).

Correlation analysis showed that *NRF2* mRNA expression was correlated with sperm parameters (sperm abnormality, total motility and DNA fragmentation). Additionally, *NRF2* gene expression was negatively correlated with MDA, while it was positively correlated with seminal plasma TAC and other antioxidant enzymes levels (including CAT, SOD and GPX) were detected both before and after NAC treatment (P<0.05 for all tests, Table 3).

**Table 2:** Comparison of biochemical factor before and after NAC


Biochemical factors	Before NAC (n=50)	After NAC (n=50)	P value

TAC(µM)	1.82 ± 0.11	2.51 ± 0.13	0.01^*^
MDA(µM)	2.36 ± 0.10	1.97 ± 0.09	0.01^*^
CAT(U/ml)	13.44 ± 2.63	18.04 ± 1.79	0.005^*^
SOD(U/ml)	0.14 ± .014	0.18 ± .006	0.01^*^
GPX(U/ml)	344 ± 12.68	378 ± 13.25	0.04^*^


Data are shown as mean ± SD, *; Significant differences between before and after NAC treatment, TAC; Total antioxidant capacity, CAT; Catalase, SOD; Superoxide Dismutase, GPX; Glutathione Peroxidase, MDA; Malondialdehyde, and NAC; N-acetylcysteine.

**Table 3:** Correlations between NRF2 mRNA level, sperm parameters and level of antioxidant enzymes before and after NAC


Correlations	NRF2	
	r	P value

Sperm abnormal morphology (%)		
Before NAC	-0.436	0.02
After NAC	-0.473	0.01
Total Motility (%)		
Before NAC	0.399	0.04
After NAC	0.499	0.01
DFI (%)		
Before NAC	-0.389	0.05
After NAC	-0.430	0.03
MDA(µM)		
Before NAC	-0.441	0.001
After NAC	-0.438	0.001
TAC (µM)		
Before NAC	0.488	0.05
After NAC	0.408	0.02
CAT(U/ml)		
Before NAC	0.226	0.05
After NAC	0.326	0.03
SOD(U/ml)		
Before NAC	0.664	0.01
After NAC	0.815	0.000
GPX(U/ml)		
Before NAC	0.194	0.094
After NAC	0.255	0.05


CAT: Catalase; DFI: DNA Fragmentation Index; GPX: Glutathione peroxidase; MDA: Malondialdehyde; NAC: N-acetylcysteine; NRF2: Nuclear factor erythroid 2-related factor 2; SOD: Superoxide dismutase; TAC: Total antioxidant capacity, and significant differences in bold.

## Discussion

The presence large number of mRNAs in human spermatozoa may effect on the events of spermatogenesis and sperm quality (18). Correlation between sperm quality and mRNA expression has previously been investigated in animals (19). Therefore Analysis of testicular genes may be an essential marker to study the role of antioxidant genes in spermatogenesis and diagnosis of male infertility.

The main results of our study revealed the role of *NRF2* gene on sperm quality through NAC supplementation *in vivo*. Enhancement of *NRF 2* gene expression by NAC may account for the improved antioxidant capacity induced by NAC. NAC is a thiol compound which can provide sulfhydryl substance. It should be taken into account that NAC has antioxidant properties. It acts via increasing the intra-cellular concentration of cysteine/GSH and scavenging free radical (20, 21). GSH plays important role in physiological functions and protection against oxidative stress (22, 23). NAC, a known antioxidant drug, can protect cells from oxidative stress through regulating *NRF2* signaling pathway by regulating GSH synthesis and maintaining the level of GSH in cells (24, 25).

Our results showed a significant improvement in the sperm parameters after 12 weeks treatment with NAC, compared to the pre-treatment baseline. The results of this study revealed that there was a relationship between *NRF2* mRNA levels and specific sperm functional parameters including, (motility, abnormal morphology and DNA fragmentation) after NAC treatment. Excessive oxidative stress directly contributed to the damage of sperm DNA by initiating apoptosis via inducing caspase-mediated enzymatic degradation of sperm DNA (26). Antioxidant administration, such as NAC, may help decrease ROS and improve sperm DNA fragmentation (27,28). A significant correlation was observed with *NRF2* mRNA expressions and sperm quality showed that the effect of NAC on sperm parameters might be mediated through *NRF2*. Several studies determined low sperm quality in humans associated with abnormal mRNA content of the certain gene (29). Yu et al. (30) showed that functional discrepancy in the *NRF2* gene promoter was correlated with abnormal spermatogenesis in humans. Previous studies showed that long term cigarette smoking can cause male infertility through inhibiting *NRF2* gene expression and sperm DNA fragmentation (31). Therefore, disruption of *NRF2* mRNA level might be one of the molecular signaling pathways of disruptive sperm function.

Defect in expression of NRF2 transcription factor is known to be critical in regulating the major determinants of the defense system against oxidative stress leading to harmful effects (32, 33). Results from the recent study demonstrated that mouse testes germ cell and Leydig cell were protected from oxidative stress in the process of heat treated-induced oxidative stress by activation of NRF2 (34). In presence of oxidative stress, NRF2 releases Keap1- mediated repression and is translocated to the nucleus. In addition, it binds to ARE located in the promoter of many antioxidant enzymes and activates the expression of AREdependent genes (35, 36). NAC acts to reduce glutathione (GSH) precursor and increasing of glutathione reductase (GR) levels by up-regulation of NRF2 expression, attenuating the ability to scavenge free radicals and oxidative stress damage (37). In this study, NAC administration increased TAC and decreased MDA levels in seminal plasma. These effects of NAC are consistent with the results obtained from previous study, indicating that NAC could improve lipid metabolism through NRF2 signaling pathway in patients with renal ischemia/reperfusion injury (38).

The obtained negative correlation between *NRF2* gene expression and MDA, in addition to the positive correlation of this gene expression with TAC suggests a possible associating effect. Previous studies reported that *NRF2*-knockout mouse had low total antioxidants levels as well as high testicular and epididymal lipid peroxidation (MDA) levels which resulted in lower sperm motility than normal males 6). According to our results, NAC significantly increased level of the antioxidant enzymes such as CAT, SOD and GPX. It was declared that there is direct correlation between NRF2 gene expression and antioxidant enzyme levels (CAT, SOD and GPX) in seminal plasma. In fact, role of *NRF 2* is to maintain homeostasis between oxidative stress and antioxidant system (37).

In contrast to these results, several studies confirmed that *NRF2* knockout decreased antioxidant genes expression and increased oxidative injury in mouse, indicating that the *NRF2*/ARE pathway is a key regulator of the body's redox state. It was reported that activity of many antioxidant enzymes (e.g. SOD and CAT) decreased in NRF2-/- mouse (39). Therefore, men with low sperm quality are likely to decrease *NRF2* mRNA and level of antioxidant enzymes. These correlations were further improved after NAC.

## Conclusion

In the present study, we observed beneficial effect of NAC, which improves sperm parameters, decreases MDA production and increases antioxidant enzyme levels, in addition to increasing NRF2 levels. Accordingly, normal human spermatogenesis requires an integrated antioxidant capability as reduced antioxidant enzyme levels may be attributed with defective sperm function. Thus, antioxidant therapy, such as NAC, may induce sperm N-Acetyl-Cysteine and *NRF2* Gene in Asthenoteratozoospermia Men 175 Int J Fertil Steril, Vol 14, No 3, October-December 2020 function by up-regulating NRF2 expression level.
